# MicroRNAs as Potential Orchestrators of Alzheimer's Disease-Related Pathologies: Insights on Current Status and Future Possibilities

**DOI:** 10.3389/fnagi.2021.743573

**Published:** 2021-10-12

**Authors:** Nermeen Z. Abuelezz, Fayza Eid Nasr, Mohammad Ahmed AbdulKader, Ahmad R. Bassiouny, Amira Zaky

**Affiliations:** ^1^Biochemistry Department, College of Pharmaceutical Sciences and Drug Manufacturing, Misr University for Science and Technology, Giza, Egypt; ^2^Department of Biochemistry, Faculty of Science, Alexandria University, Alexandria, Egypt

**Keywords:** Alzheimer's, mitochondria, microRNAs, synaptic plasticity, sleep disorder, pain

## Abstract

Alzheimer's disease (AD) is a progressive and deleterious neurodegenerative disease, strongly affecting the cognitive functions and memory of seniors worldwide. Around 58% of the affected patients live in low and middle-income countries, with estimates of increasing deaths caused by AD in the coming decade. AD is a multifactor pathology. Mitochondrial function declines in AD brain and is currently emerging as a hallmark of this disease. It has been considered as one of the intracellular processes severely compromised in AD. Many mitochondrial parameters decline already during aging; mitochondrial efficiency for energy production, reactive oxygen species (ROS) metabolism and the *de novo* synthesis of pyrimidines, to reach an extensive functional failure, concomitant with the onset of neurodegenerative conditions. Besides its impact on cognitive functions, AD is characterized by loss of synapses, extracellular amyloid plaques composed of the amyloid-β peptide (Aβ), and intracellular aggregates of hyperphosphorylated Tau protein, accompanied by drastic sleep disorders, sensory function alterations and pain sensitization. Unfortunately, till date, effective management of AD-related disorders and early, non-invasive AD diagnostic markers are yet to be found. MicroRNAs (miRNAs) are small non-coding nucleic acids that regulate key signaling pathway(s) in various disease conditions. About 70% of experimentally detectable miRNAs are expressed in the brain where they regulate neurite outgrowth, dendritic spine morphology, and synaptic plasticity. Increasing studies suggest that miRNAs are intimately involved in synaptic function and specific signals during memory formation. This has been the pivotal key for considering miRNAs crucial molecules to be studied in AD. MicroRNAs dysfunctions are increasingly acknowledged as a pivotal contributor in AD via deregulating genes involved in AD pathogenesis. Moreover, miRNAs have been proved to control pain sensitization processes and regulate circadian clock system that affects the sleep process. Interestingly, the differential expression of miRNA panels implies their emerging potential as diagnostic AD biomarkers. In this review, we will present an updated analysis of miRNAs role in regulating signaling processes that are involved in AD-related pathologies. We will discuss the current challenges against wider use of miRNAs and the future promising capabilities of miRNAs as diagnostic and therapeutic means for better management of AD.

## Introduction

### Alzheimer's: A Peculiar Case of Brain Disease

“Alois Alzheimer,” The German Bavarian psychiatrist, and neurologist was the first to report Alzheimer's disease (AD) in 1906 as “A peculiar severe disease of the cerebral cortex” (Hippius and Neundörfer, 2003). Today, Alzheimer's is acknowledged as a common neurodegenerative disease affecting elderly population. It is the most common form of dementia and may account to 60–70% of cases (Plassman et al., 2007), with increasing numbers of people getting AD every year, especially in middle and low-income countries (Alzheimer's Disease Facts and Figures, 2020).

AD is an irreversible, progressive brain disorder and the most common cause of dementia among older people. It is heterogeneous in every aspect, such as the relation between the presence of plaques and tangles of Aβ and Tau in the brain, clinical symptoms, and genetic background that causes memory loss, language problems, and impulsive or unpredictable behavior. Tau tangles block the transport of nutrients and other essential molecules inside neurons. Recently, it was found that Tau signal emerges first in the rhinal cortex independently of Aβ. Tau pathology begins focally but expands catastrophically under the influence of Aβ pathology to mediate neurodegeneration and cognitive decline. Subsequent Tau elevation in the temporal neocortex is associated with age, Aβ, and APOE status (Sanchez et al., 2021). This indicates a loss of connection between the nerve cells, or neurons, in the brain. At the earlier stages of AD, patient's daily routine is impacted due to disruptions in the entorhinal cortex and hippocampus that affects memory, executive cognition, and visuospatial awareness. Meanwhile, during later AD stages, personality, behavior, and language impairments arise due to escalating damage in frontal, temporal, and parietal lobes. Such drastic damages are associated with continuous decline in independence, culminating in patients' complete dependence on their caregivers by the latest stages of the disease. Consequently, AD is one of the most debilitating disorders as it impacts both patients and families on mental, psychological, and socio-economic aspects.

### Alzheimer's: What We Know So Far

Scientists do not yet fully understand the exact causes of AD. AD is a multifactorial disorder in which genetic and environmental risk factors interact to increase the rate of normal aging. It is so tightly associated with old age that there was a speculation it is a normal part of aging (Masters et al., 2015). Various causes probably contribute to AD etiology including a combination of age-related changes in the brain; brain proteins fail to function normally and the presence of toxic oligomeric species of Amyloid peptides (Aβ) and Tau within the AD brain. Recent data confirms the view that such species can propagate and spread within neural circuits, disrupting the work of brain cells (neurons) and triggering a series of toxic events (Chen and Mobley, 2019). Also, mitochondrial dysfunction, a compromised blood brain barrier, immune system dysfunction, and infectious agents probably contribute to the etiology of AD (Fulop et al., 2021). AD is a progressive neurologic disorder that causes the brain to shrink (atrophy) and brain cells to die, culminating into continuous decline in thinking, behavioral and social skills that affect a person's ability to function independently. Clinical manifestations, which are insidious in onset, include memory loss and cognitive decline (Scheltens et al., 2016). The most accepted theory is that AD is caused by misfolded proteins and the culprit in this misfolding is Aβ peptides, that aggregate or clump, killing brain cells and giving rise to the symptoms of memory loss and reduced cognition.

Current research identifies three stages of AD: preclinical Alzheimer's disease, mild cognitive impairment (MCI) due to Alzheimer's disease, and dementia due to Alzheimer's disease (Sperling et al., 2011). In the last two stages, symptoms are present, but to varying degrees. Early signs include difficulty remembering recent events or conversations. As the disease progresses, memory impairments worsen, and other symptoms develop.

People with MCI due to Alzheimer's disease have biomarker evidence of an Alzheimer's-related brain change (for example, elevated levels of Aβ) and greater cognitive decline than expected for their age, but this decline does not significantly interfere with everyday activities (Roberts and Knopman, 2013). In MCI, changes in thinking abilities may be noticeable to family members and friends but may not be noticeable to others.

Sadly till date, there is no cure for AD. Currently, the only approved drugs for AD merely alleviate some of the symptoms -partially and temporarily- but do not stop the disease from progressing.

### Alzheimer's Hallmarks: Aβ and Phosphorylated Tau

The neuropathology of AD is characterized by an abnormal build-up of extracellular amyloid-β (Aβ) peptide as neuritic plaques, pathological extracellular aggregates formed around a core of Aβ and are a hallmark of AD (Stratmann et al., 2016), accompanied by intracellular hyperphosphorylated (p)-Tau fibrils which accumulate as neurofibrillary tangles (NFTs) within neurons (Stratmann et al., 2016). Amyloid peptide (Aβ) is derived from the amyloid precursor protein (APP), α-secretase (“normal” cleavage), or β-secretase (“abnormal” cleavage) cleaves APP, and a second cleavage of the β-secretase product, by γ-secretase, cleaves APP further to produce Aβ (Scheuner et al., 1996; Di Carlo et al., 2012). Depending on the cleavage site by γ-secretase, Aβ40 or Aβ42 are produced. Aβ40 is the most common form, while the 42-amino-acid–long fragment, Aβ42, is less abundant and more associated with AD. The proportion of Aβ40 to Aβ42 is important in AD because Aβ42 is far more prone to oligomerize and form fibrils than Aβ40 peptide (Sun et al., 2015). Recently, Alexandra Grubman's group isolated amyloid plaque-containing (using labeling with methoxy-XO4, XO4^+^) and non-containing (XO4^−^) microglia from an AD mouse model. Transcriptomics analysis identified different transcriptional trajectories in aging and AD mice. Where XO4^+^ microglial transcriptomes demonstrated dysregulated expression of genes associated with late onset AD (Grubman et al., 2021). Recent genome-wide association studies have established that the majority of AD risk loci are found in or near genes that are highly and sometimes uniquely expressed in microglia, the resident macrophages of the central nervous system. This leads to the concept of microglia being critically involved in the early steps of the disease and identified them as important potential therapeutic targets. Changes in microglial morphology, the resident macrophages of the central nervous system, and signaling is also evident in AD brains, contributing to the pathology (Hemonnot et al., 2019).

The neuropathological changes of AD brain, based on brain imaging studies, show slow and progressive cerebral atrophy, where the frontal and temporal cortices often have enlarged sulcal spaces with atrophy of the gyri, while primary motor and somatosensory cortices most often appear unaffected (Perl, 2010). Neuropathological studies in combination of MRI approaches also showed early pathological alterations to the locus coeruleus (LC), a tiny nucleus in the brainstem and the principal site of noradrenaline synthesis. The LC undergoes significant neuronal loss in AD, with postmortem studies showing as much as 80% reduction in cell number in people with AD compared to age-matched controls associated with tauopathy in AD (German et al., 1992; Hoogendijk et al., 1995; Beardmore et al., 2021). Another macroscopic feature commonly observed in AD is the loss of neuromelanin pigmentation in the locus coeruleus with age and is proposed to be toxic and inflammatory when released into the extracellular environment (Serrano-Pozo et al., 2011).

Another characteristic feature in AD pathology is the neurofibrillary tangles of hyperphosphorylated microtubule-associated protein (Tau). Microtubules (MTs) are hollow cylinders composed of parallel protofilaments of α and β tubulin subunits. Tau is a neuronal microtubule associated protein whose main biological functions are to promote microtubule self-assembly by tubulin and to stabilize those already formed. MTs dynamics are regulated by Tau proteins that stabilize or destabilize them (van der Vaart et al., 2009) and protect MTs against depolymerization by decreasing the dissociation of tubulin at both MT ends, resulting in an increased growth rate and decreased catastrophe frequency (Trinczek et al., 1995). Disruption of microtubules, as are observed in patients with AD, interrupts axonal transport which prevents vesicles and organelles from reaching the synapses. These result in the slow and steady deterioration of the synapses and retrograde degeneration of the neurons.

### Genes of AD

Multiple studies reported different genes to be involved in AD development. Although twin studies support the existence of a genetic component in late onset Alzheimer's disease (LOAD), no one particular causative gene has been identified yet. Familial AD is mainly associated with mutations in the Aβ precursor protein (APP) gene and presenilin genes *PSEN1* and *PSEN2* that are responsible for γ-secretase cleavage of APP. Still, Familial AD comprises only the minor subclass of AD. Apolipoprotein E (APOE) on chromosome 19 is another polymorphic protein and the allele APOE4 is the strongest genetic risk factor for Sporadic AD (Giri et al., 2016). As the strong affinity of APOE for Aβ affects its production, hydrolysis, and elimination (Reitz and Mayeux, 2014). Yet, although around 80% of LOAD is associated with APOE, apolipoprotein E (APOE)-4 allele confers only a 20% risk for developing the disease. Similarly, increasing evidence confirms that Sporadic AD has a more underlying complex etiology that includes both genetic and environmental factors (Bekris et al., 2010).

The most successful approach to identifying the genetic architecture of AD is the genome-wide association studies (GWAS) which identified and confirmed 19 genome-wide-significant common variant signals in addition to APOE. Together with GWAS, Whole Exome Sequencing (WES), and Whole Genome Sequencing (WGS) defined no fewer than 20 additional genes whose variants contribute to increased risk of AD (Jun et al., 2017; Liu et al., 2017). Kunkle et al. (2019), confirmed 20 previous LOAD risk loci and identified five new genome-wide loci (*IQCK, ACE, ADAM10, ADAMTS1, and WWOX*), two of which (*ADAM10 and ACE*) were identified in a recent genome-wide association (GWAS)-by-familial-proxy of AD or dementia. Fine-mapping of the human leukocyte antigen (HLA) region confirms the neurological and immune-mediated disease haplotype HLA-DR15 as a risk factor for LOAD (Kunkle et al., 2019). Other implicated genes are Clusterin *(CLU)*, Sortilin-related receptor-1 *(SORL1)*, ATP-binding cassette subfamily A member 7 *(ABCA7)*, Bridging integrator 1 *(BIN1)*, phosphatidylinositol binding clathrin assembly protein *(PICALM)*, CD2 associated protein *(CD2AP)*, Complement component (3b/4b) receptor 1 *(CR1)*, CD33, triggering receptor expressed on myeloid cells 2 *(TREM2)*, and phospholipase D3 *(PLD3)* (Karch and Goate, 2015). These variants define possible contributions in AD from genes that regulate endocytosis, inflammation and the brain's innate immune system, and cholesterol/sterol metabolism (Karch and Goate, 2015).

### Alzheimer's Pathophysiology: Multiple Hypotheses

**Figure 1:**
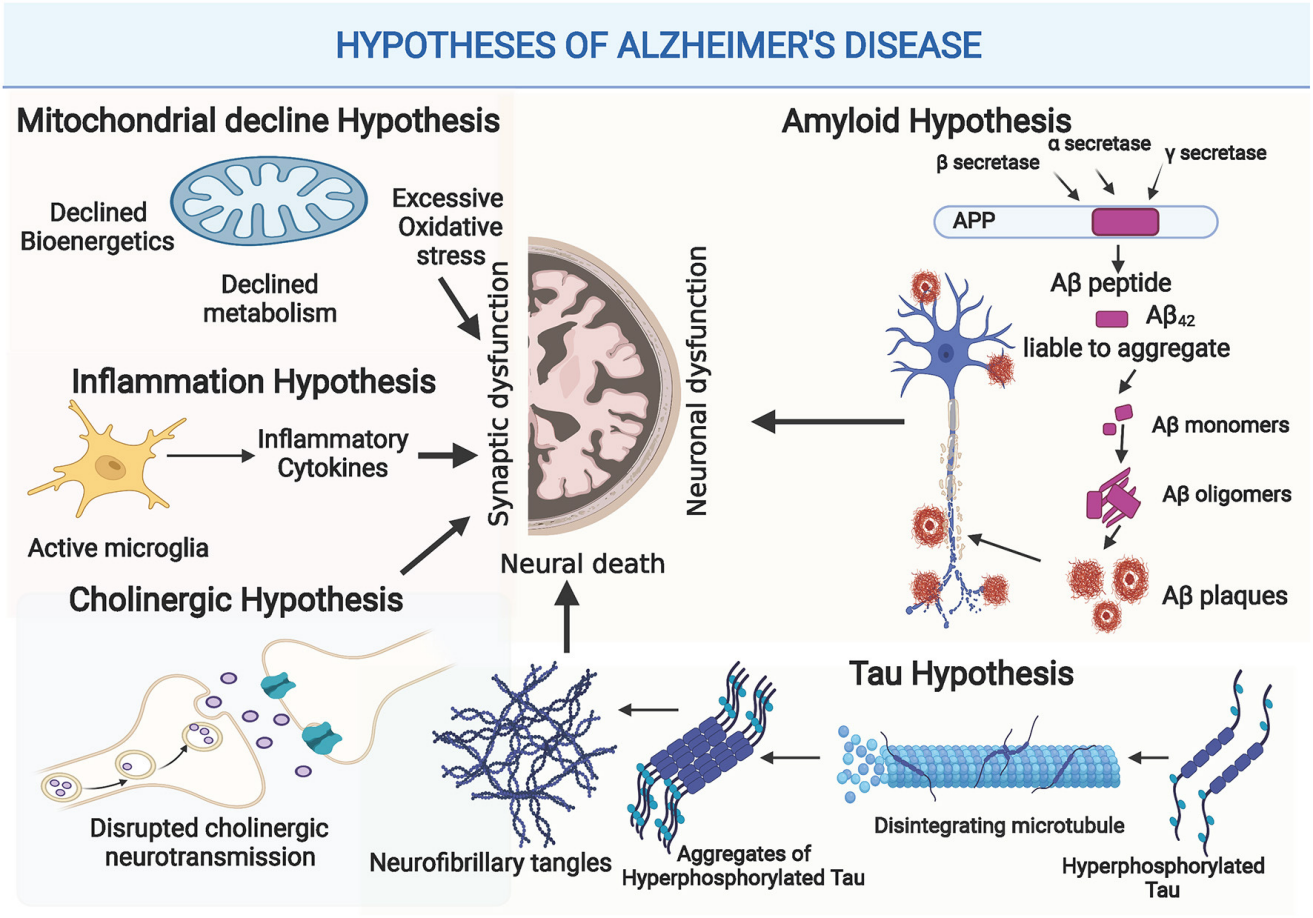
Illustration of the different hypotheses of Alzheimer's Disease (AD) Development and Progression: Amyloid hypothesis: as disturbed secretase enzymes increase production of Aβ42 eventually forming Aβ plaques that impact synaptic functionality, and neuronal dysfunction. Tau Hypothesis: Where increased production of hyperphosphorylated tau provokes disintegration of microtubules and accumulation of hyperphosphorylated tau fibrils causing neuronal death. Cholinergic hypothesis: where disturbed Acetylcholine (ACH) impacts synaptic function. Inflammation hypothesis: where increased release of inflammatory modulators provoke exaggerated immune response that disrupts synaptic functions and plasticity. Mitochondrial hypothesis: where disrupted mitochondrial functions causes glucose hypometabolism, poor ATP production and increased production of ROS, eventually causing synaptic dysfunction. (Created with BioRender.com).

The complexity of AD led to the generation of multiple hypotheses, in trials to unravel AD pathogenesis. Illustration of the different hypotheses of AD development and progression is shown in Figure 1.

### Aβ Cascade Hypothesis

For long, Aβ has been seen as the main causative agent in AD pathology that is followed by neurofibrillary tangles, vascular damage and neuronal loss as direct consequences of Aβ deposition (Murphy and LeVine, 2010). Where Aβ deposits in the hippocampus and basal segment, provoking Aβ to form insoluble aggregates, and inducing mitochondrial damage (Lustbader et al., 2004), and synaptic dysfunction (Hunt and Castillo, 2012). This cascade of events is followed by microglia and astrocytes activation, which arise inflammation and oxidative stress, causing synaptic loss and neuronal death (Fan et al., 2020). Recent studies show that elevated levels of Aβ plaque do not always correlate with magnified AD pathology. Whereas, increasing evidence reveals that Aβ oligomers (Aβ Prions) might be more neurotoxic as they are easily transmitted and released in the synapses (Mucke and Selkoe, 2012; Amin and Harris, 2021). Consequently, and considering the accumulated body of AD research, the majority of data still supports Aβ role as the principal initiator of AD complicated pathology in the early stages. Yet obviously, Aβ is not the only contributor to AD late stages.

### Tau Hyperphosphorylation Hypothesis

Tau is mainly found in neuronal axons of the brain. It maintains microtubule structure, synaptic structure, function (Kimura et al., 2014) and regulates neuronal signaling. Tau is also a phosphoprotein that depends on protein kinase and protein phosphatase activities. Hyperphosphorylated Tau in AD patients' brains causes configuration changes and loss of tubulin polymerization capacity (Grundke-Iqbal et al., 1986) that result in defective microtubule functioning. Moreover, increased levels of cytosolic Tau induce polymerization of phosphorylated Tau to form NFTs (Iqbal et al., 2010), that contribute to reduced synapses numbers, and cell dysfunction (Callahan et al., 1999). Tau hyperphosphorylation is positively correlated with the pathological severity of AD and is apparently more detrimental to cognitive impairment than Aβ (Mocanu et al., 2008).

### Cholinergic Hypothesis

The cholinergic hypothesis suggests that disruptions of acetylcholine-containing neurons contribute to the cognitive decline in Alzheimer's disease (AD). This hypothesis is supported by the fact that severity of dementia in AD, is positively correlated with the extent of cholinergic loss (Francis et al., 1985). Furthermore, multiple reports imply that cholinergic loss may be an early sign of cognitive decline in AD, and can therefore have a more crucial role in Aβ depositions, Tau phosphorylation, and neuroplasticity (Terry and Buccafusco, 2003; Hampel et al., 2018). Currently, inhibitors of acetylcholinesterase are widely used in AD management, and they show some tangible results concerning symptomatic improvement in AD patients.

### Inflammation Hypothesis

Neuroinflammation is a hallmark of AD and increasing evidence shows that microglia is a central player in AD. As in early AD stage, microglia, *TREM2* and complement system are responsible for synaptic disruptions (Paolicelli et al., 2011; Hong et al., 2016). Meanwhile, as the disease progresses, reactive microglia and astrocytes surround amyloid plaques and secrete numerous pro-inflammatory cytokines that drastically impact synaptic functions and neuroplasticity over the course of AD (Du et al., 2018).

### Oxidative Stress Hypothesis

Likewise, oxidative stress is a significant player in AD pathogenesis. Normally, the brain utilizes more oxygen than other tissues and undergoes mitochondrial respiration, which increases the potential for ROS exposure. AD is highly associated with cellular oxidative stress that contributes to increased protein oxidation, glycol oxidation, lipid peroxidation and Aβ accumulation. In line, advanced glycation end products (AGE) and malondialdehyde have been detected in the neuro tangles and senile plaques of AD (Markesbery, 1997). Moreover, multiple studies show that Aβ is capable of generating free radicals that mediate neuron degeneration and death (Cheignon et al., 2017).

### Mitochondrial Damage and Glucose Hypometabolism Hypothesis

Increasing body of evidence implies that mitochondrial impairments play fundamental roles in AD pathology. Healthy mitochondria support neuronal activity, provide proper energy supply to neurons by regulating glucose metabolism, and minimizing oxidative damage. In the neurons, mitochondria are vital for biosynthesis of iron and heme. They are also involved in presynaptic transmission, and they regulate calcium concentration during signal transduction (Picone et al., 2014).

Emerging reports spot glucose hypometabolism as an early pathogenic event in preclinical stages of AD concurrently with cognitive and functional decline. Using fluoro-2-deoxyglucose positron-emission tomography (FDG-PET), reveal that glucose hypometabolism is consistently detected in hippocampus and cortex of AD brain compared to normal individuals. In line, impaired mitochondrial bioenergetics, increased oxidative stress and disrupted mitochondrial genome are consistent features of mitochondrial abnormalities in AD and are interconnected to amplify the debilitating pathologies of AD (Wang W. et al., 2020).

Till date, it is not totally clear whether any of these characteristic deficits is the primary initiator or just a contributor in the contemporary multifactorial AD pathology. A challenge confirmed by the little therapeutic success achieved against AD so far. Consequently, we obviously need new approaches for AD diagnosis at its earliest stages before neuronal damage becomes irreversibly established. It is also crucial to concurrently target multiple axes in AD pathologies for more tangible therapeutic feasibility.

### MicroRNAs: Origin, Maturation, and Role

MicroRNAs production excels with formation of a long primary transcript (pri-miRNA) via RNA polymerase II. Inside the nucleus, pri-miRNAs are cleaved by Drosha protein and DiGeorge Syndrome Critical region 8 (DGCR8) protein which dimerize together to form a functional microprocessor complex. These dimerized proteins cleave pri-miRNAs into precursor miRNAs (pre-miRNA), which are then transported to the cytoplasm and digested by Dicer and TAR RNA binding proteins (TRBP) to release a double-stranded miRNA duplex. Helicase enzyme unwinds the duplex to form mature miRNA strands. One of these strands is usually degenerated, while the other associates with Ago2 protein to form miRNA-induced Silencing Complex (miRISC). Each mature miRNA contains a sequence of 7 or 8 nucleotides that binds to its complementary region(s) on target mRNAs. Mature miRNAs bind to the 5′- or 3′-untranslated regions (UTR) of target mRNAs and rarely, both strands can serve as mature functional miRNA. Generally, functional miRNAs induce gene silencing using two different mechanisms, depending on the complementarity between the miRNA and its mRNA target (Amakiri et al., 2019; Kou et al., 2020). If mature miRNA binds perfectly to the complementary regions of the target mRNA, it induces mRNA degradation via de-adenylation, cap removal, and exonucleolytic digestion of mRNA. Meanwhile, if miRNA binds with imperfect complementarity to target mRNA, it causes a translation block by repression of translation during the initial phase or the elongation phase. Alternatively, miRNAs can repress translation by inducing premature ribosome detachment (Silvestro et al., 2019). One functional miRNA can interact with hundreds of target mRNAs to exert various levels of regulatory effects, and a single mRNA can be targeted by several miRNAs as well.

Around 70% of miRNAs are found in the human brain, where miRNAs are responsible for regulating synaptic functions, neurotransmitter release, and neuronal development. In the last few years, the significance of balanced miRNAs expression proved to be crucial for proper functionality and homeostasis in the body. The differential expression of miRNAs and/or single nucleotide polymorphism (SNP) of miRNAs are implicated in multiple diseases (Reddy et al., 2017). Likewise, the dysregulation of miRNAs emerged as a key contributor in AD pathology, as it leads to altered protein expressions and impairment of the complicated signaling network balance in the brain. The next section will cover the main updated findings concerning miRNAs role in AD pathogenesis and its progressive events.

## Micrornas and AD Cognitive Impairment

**Figure 2:**
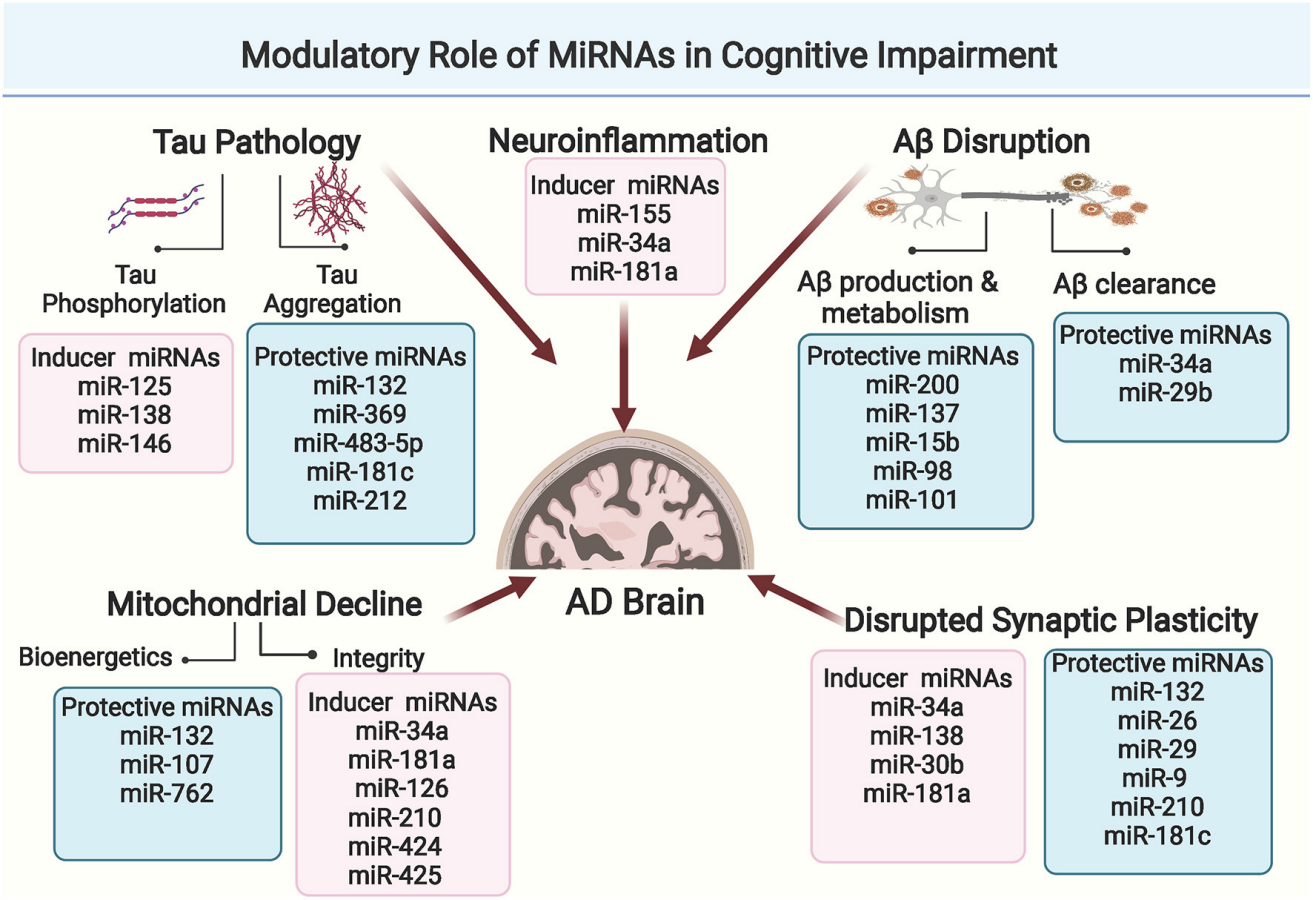
Comprehensive illustration of the modulatory role of miRNAs in cognitive impairment in AD through affecting Aβ and Tau metabolism, mitochondrial functionality, neuroinflammation or synaptic plasticity. (Created with BioRender.com).

Cognition refers to the brain's ability to think, learn, remember, and process information. The loss of neuronal connections in AD brain is basically attributed to disrupted signaling pathways that affect both synaptic plasticity and dendritic functions, the two crucial controllers of cognitive processes. On the molecular level, updated studies show that Aβ and Tau pathologies cause progressive axonal degeneration and drastic downstream impairments in the synaptic processes (Pereira et al., 2021). Moreover, Aβ and Tau aggregates induce exacerbated immune microglial response, as well as disrupted astrocytes functionality, that eventually contribute to cognitive decline in AD (Fakhoury, 2018). Over the past few years, miRNAs have emerged as significant regulators of Aβ and Tau metabolism, glial functionality, and synaptic plasticity. Simultaneously, increasing studies report the drastic impact of miRNA dysregulations on cognitive functions in AD, through targeting key genes and activity-mediated protein synthesis at the synaptic level. Comprehensive illustration of the modulatory role of miRNAs in AD-related cognitive impairment is presented in Figure 2.

### MicroRNAs and Aβ Pathway Disruptions

Although amyloid deposition alone is not able to produce full AD-pathology, studies that used Aβ PET in cognitively normal elderly individuals, mild cognitive impairment (MCI) and AD patients found significant relationships between cognitive deficits and increased brain fibrillar Aβ (Wang F. et al., 2015). MicroRNAs are closely related to the synaptic dysfunction induced by abnormal Aβ metabolism. Increasing body of work shows that cognitive impairment caused by Aβ can be restored by manipulation of miRNAs, which strongly supports the belief that disrupted miRNA expressions are critical in cognitive impairment in AD patients (Weldon Furr et al., 2019). MicroRNA dysregulations are repeatedly reported in association with key genes that regulate Aβ synthesis, cleavage, and clearance. Table 1 illustrates an updated comprehensive summary of the human studies showing differentially expressed miRNAs in AD samples and their relation to Aβ pathway regulations.

**Table 1:** Disturbed miRNAs reported in human AD studies and their relation to Aβ pathways.

**miRNA**	**Sample**	**Regulation**	**Relevant pathway**	**References**
miR-15a miR-15 b	Serum	Downregulation	Downregulates BACE1 expression	Satoh et al., 2015; Lang et al., 2016; Li and Wang, 2018
miR-29a	Brain CSF Serum	Down Up Down	BACE1	Hébert et al., 2008; Geekiyanage et al., 2012; Müller et al., 2016
miR-29b	Blood	Down	BACE1	Satoh et al., 2015
miR-29c	Brain	Down	BACE1	Lei et al., 2015
miR-20b	Brain	Down	APP	Nunez-Iglesias et al., 2010
miR-21-5p	Brain	Up	APP	Giuliani et al., 2021
miR-16	CSF	Down	APP	Müller et al., 2014
miR-9	Brain	Down	APP	Hébert et al., 2008
miR-30c	Brain	Down	APP	Cogswell et al., 2008
miR-101a-3p	Brain	Down	APP	Hébert et al., 2008
miR-188	Brain, Serum	Down	APP	Kou et al., 2020
miR-106a	Brain Serum	Down Up	APP	Wang et al., 2011; Cheng et al., 2015
miR-106b	Brain Whole blood	Down Up	APP	Hébert et al., 2008; Cheng et al., 2015
miR-138-5p	Plasma Exosomes	Up	APP	Lugli et al., 2015
miR-138	Brain, CSF	Variant	APP	Boscher et al., 2019
miR-135b	Blood	Down	BACE1	Zhang et al., 2016
miR-124	CSF	Down	APP	Burgos et al., 2014
miR-155	Peripheral Blood mononuclear cells	Upregulated	Inhibits Aβ catabolism	Guedes et al., 2016
miR-34	Peripheral Blood mononuclear cells	Upregulated	Aβ clearance	Basavaraju and de Lencastre, 2016
miR-181b	Peripheral Blood mononuclear cells	Upregulated	Aβ clearance	Kumar and Reddy, 2016
miR-181c	Serum	Downregulated	APP	Geekiyanage et al., 2012
miR-186	Brain	Downregulated	BACE1	Ben Halima et al., 2016
miR-346	Human Neuron Enriched culture	Upregulated	APP	Long et al., 2019
miR-338	Hippocampus	Downregulated	APP	Qian et al., 2019
miR-200	Brain	Upregulated	SIRT1, Aβ accumulation, Apoptosis	Zhang et al., 2017

Regarding Aβ metabolism, several miRNAs including miR-9, miR-29, miR-135, and miR-186 are significant regulators of Beta-Secretase 1 (*BACE1)* enzyme levels which is central in Aβ generation (Wang et al., 2019).

MiR-200-3p particularly has been grabbing increased attention for its role in Aβ pathology in AD. MiR-200-3p is repressed in AD animal and cell models. Mechanistically, miR-200-3p modulates translocation of BACE1 enzyme and ribosomal protein S6 kinase B1 (S6K1), hence it suppresses cell apoptosis, decreases Aβ1-42 and Tau phosphorylation in cell experiments (Samadian et al., 2021). To evaluate the effect of miRNA-200b/c *in vivo*, Tg2576 mice were treated with miRNA-200b/c by intracerebroventricular injection. This experiment confirmed that upregulating miR-200 reduced secretion of Aβ. Moreover, the treated mice were relieved of memory impairments induced by intracerebroventricular injection of oligomeric Aβ. They also demonstrated proper spatial learning, suggesting that miRNA-200b and miRNA-200c are potential therapeutic targets in AD (Higaki et al., 2018). These data are strongly supported by clinical studies that showed decreased miR-200b in serum and cerebrospinal fluid of AD patients, compared to healthy subjects (Silvestro et al., 2019).

Recent studies have recently shown that miR-137 and miR-15 b can reverse the neurotoxicity induced by Aβ abnormal metabolism in animal and cell lines (He et al., 2017; Li and Wang, 2018). In the updated pilot study of Vergallo et al. (2021), the protective anti-Aβ effect of miR-15b is reported in asymptomatic at-risk population for AD, as there were significant associations between plasma concentrations of miR-15b, with core neuroimaging biomarkers of AD pathophysiology in the hippocampus (Vergallo et al., 2021).

Several miRNAs as miR-98 and miR-124 modulate Aβ production via notch signaling pathway. Specifically, miR-98 suppresses Amyloid accumulation as it inhibits *HEY2* protein levels which inactivates the notch signaling pathway responsible for Aβ production (Amakiri et al., 2019).

Furthermore, miRNAs that directly target *APP*, confirm the role of miRNAs in AD pathogenesis. Downregulated levels of miR-101 are reported in AD brain and are consistent with *in vitro* studies where inhibition of miR-101 increased APP levels (Siedlecki-Wullich et al., 2021). *In vitro* and *in vivo* studies also show that downregulation of miR-137 determines an increase in Ca2^+^ levels and a reduction of Aβ1-40 and Aβ1-42. These results indicate that an increase in miR-137 could cause a decrease in Ca2^+^ levels in neurons, improving neuronal dysfunctions of AD (Davare and Hell, 2003).

Another approach of AD Aβ pathology is the Aβ clearance from the brain to the circulation. Aβ clearance from the brain requires adequate balance of Aβ phagocytosis, glymphatic clearance and healthy system of ABC transporters *ABCB1* and *ABCG2*. Recently, miR-34a and miR-29b have been found to interfere with at least three pathways of Aβ clearance (Weldon Furr et al., 2019). In adult mammalian brain, miR-34a and miR-29b are highly expressed and have been implicated in a range of neurodevelopmental and neuropathological processes. Both miR-34a and miR-29b are dysregulated in brain and serum samples of AD patients (Madadi et al., 2019).

Taken together, these cumulative studies show that AD disease can disrupt miRNA coordinated expression. Simultaneously, miRNA altered expression contributes to Progressive AD pathogenesis through disrupting key genes in Aβ pathway. Unleashing the mechanism of microRNA Aβ regulating pathways, can identify novel therapeutic targets for better AD management.

### MicroRNAs and Tau Pathology in AD

In the last few years, abnormal phosphorylated Tau has proved to be detrimental in cognitive decline (Di et al., 2016). Moreover, extracellular soluble Tau oligomers have been recognized as a possible cause of memory loss and synaptic dysfunction (Biundo et al., 2018). Experimental AD studies helped to identify several miRNAs that are linked to Taupathy in AD. Among the most prominent miRNAs, miR-125b, and miR-138 are upregulated in AD and have been shown to induce Tau hyper phosphorylation and tangling in neuronal cultures. Subsequently their upregulation disrupts associative learning and cognition in AD mice models (Banzhaf-Strathmann et al., 2014; Wang X. et al., 2015). MiR-125 upregulation in AD promotes Tau hyperphosphorylation through activating Mitogen-activated protein kinase (*MAPK)* kinases, most likely by down-regulating its target phosphatases genes: *DUSP6* and *PPP1CA*. Whereas, direct hippocampal delivery of miR-125b mimic improved learning, memory and inhibited Tau phosphorylation and expression of DUSP6, and PPP1CA in C57BL/6 mice (Banzhaf-Strathmann et al., 2014). Other miRNAs are reported to modulate Tau affinity for microtubule, regulate the maintenance of microtubule network, and affect Tau aggregation/deposition in NFTs (Siedlecki-Wullich et al., 2021). MiR-22-3p affects Tau phosphorylation through regulating Sirtuin 1 *SIRT1* gene. Meanwhile, miR132-3p regulates Tau phosphorylation via *PTBP2* gene and Tau splicing via modulating *MeCP2* and *PTEN* genes (Praticò, 2020). In 3xTg mice, loss of miR-132 increased total and phosphorylated Tau levels and provoked Tau aggregation. Consistently, restoring miR-132 to normal levels improved Tau pathology and long-term memory (Smith et al., 2015). Similarly, miR146a-5p is reported to regulate Tau phosphorylation via *ROCK1* gene.

Clinically and in support of these translational experiments, miR-125, miR-138, miR-146 and miR- 132 are significantly dysregulated in the cerebrospinal fluid of AD patients (Galimberti et al., 2014; Lee et al., 2016; Wei et al., 2020). MiR-369 is one of the most recently studied miRNAs. Knocking out miR-369 in 3xTg AD mice aggravated cognitive impairment and promoted hyperphosphorylation of Tau, through upregulating kinases *Fyn* and serine/threonine-protein kinase 2 *(SRPK2)* as the upstream molecules. Meanwhile, Restoring miR-369 reversed the hyperphosphorylation of Tau and downregulated *Fyn* and *SRPK2*, implying the possible therapeutic potential of miR-369 in AD (Yao et al., 2020).

Similarly, miR-483-5p is recently reported to regulate *ERK1* and *ERK2* kinases at both mRNA and protein levels, resulting in reduced phosphorylation of Tau protein associated with Tau neurofibrillary pathology in AD. Taking these observations together, suggests the neuroprotective action of miR-483-5p in AD pathology (Nagaraj et al., 2021).

Besides Tau phosphorylation, miRNAs have a key role in Tau clearance. Where some post-translational modifications of Tau inhibit Tau ubiquitin binding which promote Tau aggregation. Moreover, acetylation at specific sites of Tau provokes autophosphorylation, and aggregation. This acetylation process is dependent on the balance between acetyltransferase p300 (an acetylase) and sirtuin 1 (SIRT1, a deacetylase). Recent studies demonstrated that *SIRT1* gene could be directly inhibited by miR-9, miR-212, and miR-181c to imply their potential role in Tau regulation and consequent AD events (Zhao et al., 2017; Praticò, 2020).

### MicroRNAs and Synaptic Plasticity in AD

Dendrites and dendritic spines are the loci of long-term synaptic plasticity that facilitates cognitive processes such as learning and memory. On the cellular level, synaptic plasticity is mediated by structural changes and functionality of dendritic spines. The dendritic spines have specialized subdomains that contain scaffolding proteins, signal transduction molecules, ion channels, and cytoskeleton components which collectively regulate spine morphology, synaptic transmission, and plasticity. Assembly and remodeling of neuronal circuit are generally affected by alterations in density and properties of ionic channels and proteins of the dendritic spines (Bosch and Hayashi, 2012; Reza-Zaldivar et al., 2020).

Growing evidence confirms that alterations of spine morphology and dendritic spine (DS) loss are correlated with AD cognitive decline even before neuronal loss (Dorostkar et al., 2015). Furthermore, the characteristic Aβ and Tau pathologies in AD, suppress synaptic plasticity, which simultaneously provokes changes in dendritic morphology, synaptic maturation and synaptic loss (Pereira et al., 2021). Moreover, updated postmortem examination of AD brains shows that cognitive impairment correlates with synaptic loss better than the number of extracellular plaques or NFTs in AD, making synaptic failure a hallmark of AD (Kumar and Reddy, 2020).

**Figure 3:**
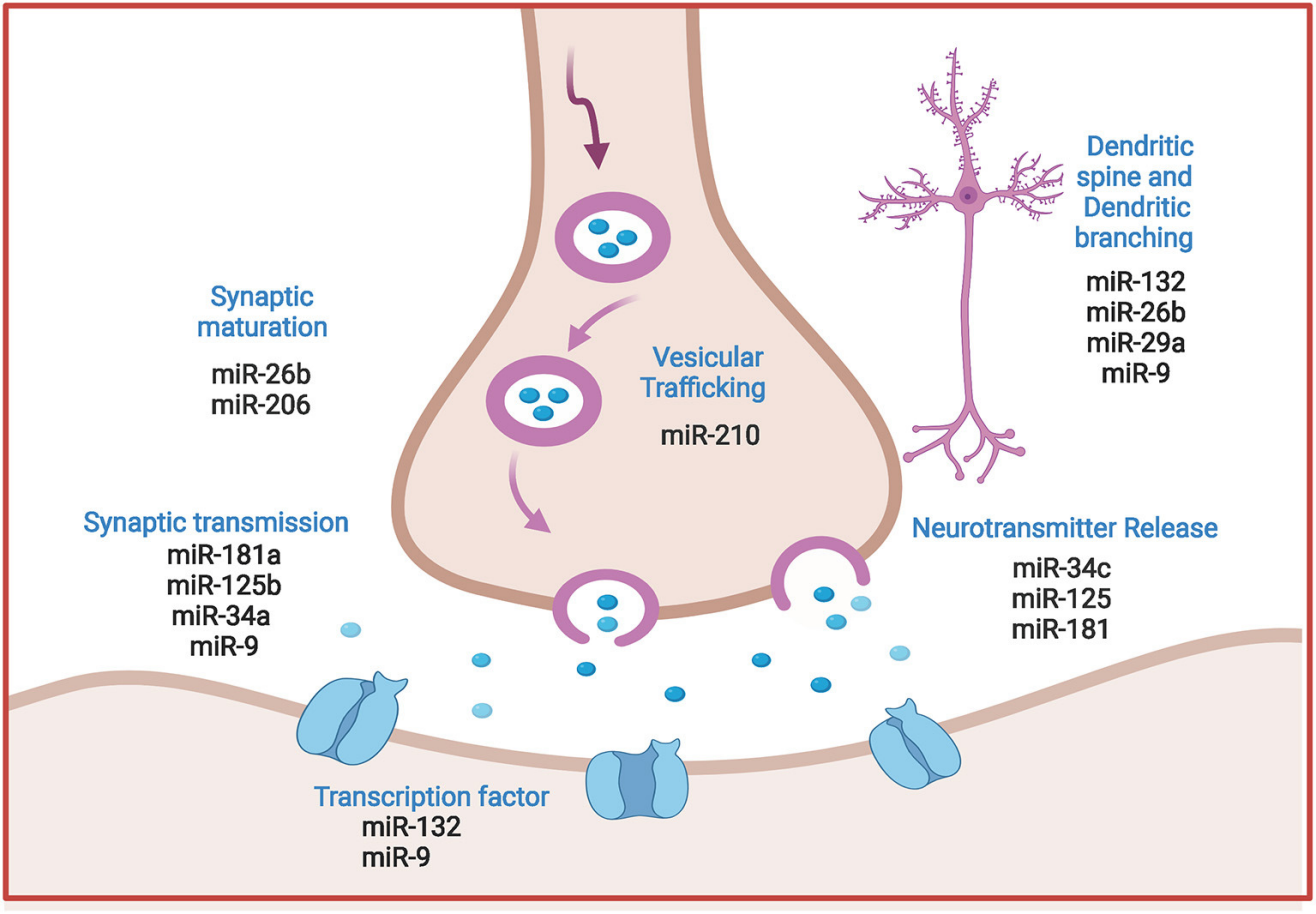
Comprehensive illustration of the regulatory role of prominent miRNAs in synaptic plasticity in AD. MicroRNAs regulate vesicle formation and trafficking, ACH release and neurotransmission, post synaptic transcription processes and dendrites morphology. (Created with BioRender.com).

Interestingly, microRNAs are now established as principal regulators of synaptic plasticity during neuronal circuit formation and integration. Moreover, changes in neuronal microRNA expression contribute to synaptic function modification via modulating dendritic spine morphology and/ or regulating local protein translation to synaptic transmission. These mechanisms are proved determinant for both synapse formation and synaptic plasticity (Reza-Zaldivar et al., 2020). Figure 3 spots the prominent regulatory role of miRNAs in synaptic plasticity.

Multiple studies reported miRNAs that contribute to impaired synaptic plasticity in AD. Table 2 illustrates an updated summary of the miRNAs involved in regulating target genes of synaptic functions, morphology and dendritic spine alterations in AD studies. Among the interesting miRNAs, miR-34a overexpression is found to impact synaptic functionality and cognitive decline in AD mice models (Sarkar et al., 2019). On the molecular level, miR-34a targets *SIRT1, CREB* and *BDNF* genes that have multiple roles in AD progression via increasing Tau Phosphorylation, altering spine morphology and spine functions. Loss of miR-101 in hippocampal neurons was found to cause cognitive decline and modulation of AD-related genes in mice. Where miR-101 knockdown in the hippocampus of C57BL/6 J mice showed AMPK hyperphosphorylation, upregulation of miR-101 target genes associated with AD such as *APP*, and *Rab5* and overproduction of Aβ42 levels (Barbato et al., 2020).

**Table 2:** MicroRNAs involved in modulating key target genes of synaptic plasticity in AD.

**miRNA**	**Target gene**	**Function**	**References**
miR-34a	*GRIN2B* *Syt-1* *Stx-1A*	Synaptic transmission reduced dendritic trees and alteration of DS morphology and function	Agostini et al., 2011; Xu et al., 2018; Sarkar et al., 2019
miR-210	*NPTX1 AMPAR*	Recruitment/clustering, synaptic transmission	Pulkkinen et al., 2008
miR-125b	*GRIN2A* *EPHA4*	Synaptic transmissionFormation of long and narrow DS filopodia-like, with a subsequently synaptic transmission weakening	Alsharafi et al., 2016 Edbauer et al., 2010
miR-181a	*GRIA2*	Synaptic transmission	Rodriguez-Ortiz et al., 2020
miRNA-574	*Nrn1*	DS stabilization leading to cognitive impairment	Li F. et al., 2015
miRNA-29a/b	*Arpc3*	Reducing the mushroom-shaped DS formation, and DS head enlargement, a fundamental step in synaptic maturation	Lippi et al., 2011
miRNA-135	*CPLX1 CPLX2*	Impairment of the postsynaptic exocytosis of AMPA receptors leading to DS shrinkage	Hu et al., 2014
miRNA-124	*CREB*	Serotonin-induced long-term synaptic plasticity	Rajasethupathy et al., 2009
miR-128	*STIM2*	Negative modulator of intracellular calcium	Deng et al., 2020
miR-92, miR-137, miR-501	*GRIA1*	Synaptic transmission	Letellier et al., 2014; Hu et al., 2015; Olde Loohuis et al., 2015
miR-9	*REST*	Synaptogenesis, synaptic plasticity, and structuralRemodeling	Giusti et al., 2014
miR-132, miR-134	*CREB1* *MMP-9* *BDNF*	Transcription factors involved in synaptic plasticity Alterations in DS morphology and maturation	Mellios et al., 2011; Jasińska et al., 2016
miRNA-206	*BDNF*	Decreasing DS density	Lee et al., 2012
miRNA-218	*GRIA2*	Increasing the amplitude of synaptic currents and formation of thin DS	Rocchi et al., 2019
miRNA-30b	*EphB2, SIRT1, GRIA2*	Reduced basal synaptic transmission, impaired spatial learning and memory retention and DS density reduction	Song et al., 2019

Similarly, overexpression of hippocampus miR-30b disrupts basal synaptic transmission, and reduces DS density, eventually leading to declined learning and memory (Song et al., 2019). This is accomplished as miR-30b modifies SIRT1 expression which regulates Tau phosphorylation. Interestingly miR-30b also targets *EphB2* that has a protective role against Aβ oligomers accumulation and disruption of glutamate receptors that are directly linked to synaptic plasticity and cognitive processes (Cissé et al., 2011). Another crucial target of miR-30b is the *GRIA2* gene, the predominant excitatory neurotransmitter receptors in mammalian brain (Siedlecki-Wullich et al., 2021), and an increasingly reported linker between Aβ clearance and synaptic disruptions in AD models (Hettinger et al., 2018).

On the clinical side, miR-138 is highly expressed in the dendrites of hippocampal neurons and it acts to regulate dendritic spine size and structure. Functional screening demonstrates that Acyl Protein Thioesterase1 (APT1)-induced palmitoylation of G protein α13 (*G*α*13*) is important for the regulatory function of miR-138 during dendritic spine development. Whereas, high level of miR-138 significantly reduces APT1 level which leads to dendritic spine shrinkage and concomitant reduction in synaptic transmission (Siegel et al., 2009). Recently a panel of studies focusing on miR-138 function during the process of learning and memory showed a close association with local plasticity-related protein synthesis. Examination of human postmortem brain tissue showed expression of miR-138 and decapping mRNA 1B *(DCP1B)* in hippocampus and frontal cortex. Furthermore, it was found that a human memory-associated single nucleotide polymorphism could interfere with miR-138 binding to the transcripts of DCP1B, implying that miR-138 is a strong modulator of human memory performance (Ye et al., 2016).

Contrarily, some miRNAs have protective effects against synaptic disruptions in AD. For instance, miR-132 inhibits extra synaptic gene Matrix metallopeptidase 9 *(MMP-9)*, whose overexpression promotes formation of immature DS. Consequently, MMP-9 inhibition by miR-132 supports DS head widening that potentiates synaptic plasticity (Jasińska et al., 2016). In line, a recent study showed that upregulating miR-132 by an enriched environment, enhanced hippocampal synaptic plasticity and prevented DS impairments induced by Aβ oligomers (Wei et al., 2020). Furthermore, miR-26a and miR-384-5p have been found as significant regulators of dendritic spine growth and targeting endogenous ribosomal S6 kinase 3 (*RSK3*). Inhibition of miR-26a is reported to attenuate neurite outgrowth and neuronal morphogenesis (Gu et al., 2015).

Collectively, these data spot the significant role of miRNAs as key players in synaptic plasticity and the impact of miRNAs dysregulations on synaptic homeostasis and functionality in AD pathology. Getting deeper understanding of miRNAs and their targets concerning synaptic modeling in AD may provide new approach to earlier diagnosis and therapeutic management of AD cognitive pathology.

### MicroRNAs and Glia Cells Role in AD-Cognitive Impairment

Microglia are brain-resident myeloid cells that mediate innate immune responses in the CNS. Under normal conditions, microglia exist in a “resting” state where they “monitor” the surrounding microenvironment and maintain brain homeostasis via synapse organization, removal of debris by phagocytosis and release of neurotrophic factors (Fan and Pang, 2017). Meanwhile, activation of microglia is accompanied by morphological changes that permit motility and phagocytosis. Microglia can differentiate into either M1 (pro-inflammatory) or M2 (anti-inflammatory) phenotypes depending on the provoking signals. M2 microglia release anti-inflammatory and protective cytokines such as IL-10, TGF-β, IL-4, and IL-13, which promote repair (Guedes et al., 2013). Whereas, M1 microglia release inflammatory mediators such as ROS, MMP-9 and pro-inflammatory cytokines such as TNFα, IL-6 and IL-1β.The balance between these different microglial phenotypic states promotes inflammation or tissue repair and influences the progression of neuroinflammatory disorders (Guedes et al., 2014).

Currently, “Microglia” is an increasing hot topic in AD research, and cumulative studies spot the multifaceted role of microglia as beneficial or detrimental in AD. Recent genome-wide association studies confirm that most of AD risk loci are present in or close to genes that are highly and/or uniquely expressed in microglia. This strongly implies the significant involvement of microglia in early steps of AD (Hemonnot et al., 2019). Among the well-known genes are *Cd33* and *TREM2* which are linked to Aβ phagocytosis and regulation of microglial inflammatory interaction with Tau tangles (Onuska, 2020).

Additionally, the CX3CR1 receptor is predominantly expressed in microglia. Its ligand CX3CL1 is constitutively expressed by neurons, and it helps maintaining microglia in a resting state. CX3CL1-CX3CR1 is a critical signaling pathway that is disrupted in neurodegenerative conditions and is associated with a strong microglial toxicity (Keren-Shaul et al., 2017). The involvement of the CX3CL1/CX3CR1 signaling pathway in AD is confirmed by an elevated plasma concentration of CX3CL1 in AD patients compared to healthy control subjects.

Other interesting contributors are the complement proteins C1q and CR3 (Veerhuis et al., 2011) which are highly produced in the microglia as crucial factors in synapse pruning and are highly present in CSF of AD patients with emerging evidence about their role in Aβ pathogenesis (Fatoba et al., 2021). Furthermore, Numerous studies demonstrate that microglial Aβ phagocytosis contributes to degeneration by triggering NLR family pyrin domain containing 3 receptor *(NLRP3)* and lysosomal cathepsin-B that subsequently releases IL-1β and disrupts autophagosome degradation.

Growing body of evidence shows that miRNAs dysregulation impacts microglial hyper-activation, neuroinflammation, and alters macrophage polarization in the brain. Mechanisms that are closely implicated in AD pathology. Table 3 illustrates updated data about miRNAs that regulate key genes in the microglia.

**Table 3:** MicroRNAs and their key target genes in the microglia.

**miRNA**	**Target Gene**	**Gene function**	**References**
miR-124	*C/EBP-α*	Microglial Polarization to M2	Ponomarev et al., 2011
miR-155	*C-MAf, CCM1, NOX2, NOX4*	Microglial polarization to M2, microglial activation and increased count	Guo et al., 2019
miR-9	*NF-kB*	Microglial activation	Yao et al., 2014
miR-204	*SIRT1*	Repression of microglial activation	Li L. et al., 2015
miR-125b	*NF-kB*	Contributing in increased TNF-α release	Parisi et al., 2013
miR-34a	*TREM2*	Aβ42 aggregation and accumulation and microglial activation	Alexandrov et al., 2013

Concerning AD studies, miR-34a is reported as a major target of *TREM2*, a microglial receptor that mediates Aβ42 clearance via phagocytosis in the CNS. Simultaneously, multiple clinical reports showed miR-34a level is dysregulated in AD patients (Bhattacharjee et al., 2016).

In line, both miR-155 and miR-146a are upregulated in the CNS during AD and both regulate the excessive inflammatory signaling observed in AD disease course (Su et al., 2016). MiR-155 is now established as a crucial pro-inflammatory factor in microglia, as it represses suppressor of cytokine signaling 1 gene (*SOCS-1*). Increased miR-155 expression was recently reported in 3xTg AD mice brains (Guedes et al., 2014), together with enhanced microglial activation. Meanwhile, knockdown of miR-155, induced *SOCS-1* expression and led to downregulation of iNOS and nitric oxide production.

MiR-146 is another multi-faceted miRNA that is implicated in the pathogenesis of AD (Jayadev et al., 2013). In microglia, miR-146 is reported to target Presenilin 2 (*PS2*), a membrane associated protease that regulates proinflammatory microglial behavior (Wang and Wang, 2018).

Astrocytes are another class of glial cells that affect inflammatory response in the CNS. In healthy conditions, astrocytes regulate neuronal metabolism, synaptogenesis, intracellular calcium levels and interact with neuronal signaling (Vasile et al., 2017). Under pathological conditions, astrocytes participate in shaping the CNS response to stress and disease. Where neuroinflammation can be either promoted or restricted by astrocytes through release of pro-inflammatory or anti-inflammatory molecules, leukocyte recruitment and forming functional barriers for CNS parenchyma (Sofroniew, 2015).

In the brain of AD patients, the inflammatory response aggravates astrocytes number, volume and activity (Meraz-Rios et al., 2013). Interestingly, IL-1β, IL-6, and transforming growth factor-β (TGF-β) are upregulated before Aβ aggregation and Tau hyperphosphorylation. These Inflammatory factors activate astrocytes to over express BACE1 enzyme and produce excessive amounts of Aβ proteins (Blasko et al., 2000). In turn accumulated Aβ provokes astrocytes to release more cytokines as TNF-α, a crucial factor in AD-related cognitive impairment (Veeraraghavalu et al., 2014). Additionally, astrocyte dysfunction leads to a decrease in Aβ uptake and clearance (Rolyan et al., 2011). Moreover, astrocytes have been recently reported to promote Tau lesions and accelerate NFTs formation (Birch et al., 2014; Yang et al., 2019).

Increasing reports spot the disruption of miRNA expression in astrocytes during neuroinflammation and neurodegenerative processes accompanying AD. Among the characteristic miRNAs is miR-146 a. Cui et al. (2010) found that miR-146a was upregulated in human astrocytes when exposed to Aβ. The study found that miR-146a mediated down-regulation of interleukin-1 receptor-associated kinase-1 (IRAK-1). IRAK-I is coupled to NF-κB extensive sustained inflammatory response of astrocytes and downstream Toll like receptors proteins. Therefore, NF-κB inhibitors and miR-146a can present a treatment strategy against excessive immune response to Aβ in the brain (Cui et al., 2010).

Glutamate release, reuptake, and recycling are tightly regulated by astrocytes at tripartite synapses. Glutamate overload can trigger neuronal and synaptic loss (Marttinen et al., 2018). Glial glutamate transporter 1 (GLT-1) contributes to clearance and regulation of glutamate at synaptic clefts. In a 3xTg- AD mouse model, increased levels of miR-181a downregulated synaptic proteins related to GLT-1, impacting the plasticity of glutamatergic synapses in astrocytes, and implying its key mediating role in synapses plasticity (Zumkehr et al., 2015). Generally, miR-181 family regulates neuroinflammatory signaling in astrocytes, and miR-181 family has been reported to be upregulated in AD mouse model, causing impaired synaptic plasticity through targeting *SIRT-1*.

MiR-155 is another significant astrocyte modulator, as it affects astrocytes density during inflammation. Furthermore, increased astrocytes levels of miR-155 were shown to target *SOCS1*, a negative regulator of the inflammatory gene response, in Aβ-treated astrocytes, causing prolonged expression of inflammatory cytokines (Guedes et al., 2014).

### MicroRNAs and Mitochondrial Damage

A large body of research shows enormous mitochondria alterations in the brains of AD patients. Interestingly, mitochondrial alterations have been consistently observed before the clinical onset of AD (Swerdlow, 2018). As a result, mitochondrial dysfunction is now a hot topic in AD for its possible role in the earlier progression of the disease.

One important feature of mitochondrial alterations in AD is the impaired mitochondrial bioenergetic machinery. Glucose hypometabolism in AD brain is strongly linked to impaired oxidative phosphorylation. Moreover, it is closely correlated to impaired levels of blood thiamine diphosphate (TDP), a crucial coenzyme of pyruvate dehydrogenase and α-ketoglutarate dehydrogenase (KGDHC) enzymes allocated in the Krebs cycle (Sang et al., 2018).

Furthermore, Redox proteomics studies found that many antioxidant enzymes that are allocated in the mitochondria, including glutathione-S-transferase Mu, peroxiredoxin 6, GSH and ATP synthase are oxidized in AD, which might compromise their functions by increasing oxidative stress conditions that prevail in AD affected brain regions (Swomley and Allan Butterfield, 2015). Interestingly, levels of oxidized nucleic acids in mtDNA are reported to be significantly elevated in preclinical AD, again stressing mitochondrial abnormalities as an early event of AD progression (Wang W. et al., 2020). Additionally, emerging studies demonstrated impaired base-excision repair (BER) activity in both AD and MCI patients (Lillenes et al., 2016), suggesting significant contribution of replication error to increased mtDNA mutations in AD.

Only recently, a subset of microRNAs is found to be localized to human mitochondria (mitomiRs) and while mitomiRs functions are still far from being completely explored, recent findings relate mitomiRs to neurodegenerative diseases, including Alzheimer's. MiR-107 is one of the recently discovered mitomirs. It regulates oxidative abilities of mitochondria and downregulated miR-107 was found to decrease mitochondrial volume, cristae and mitochondrial membrane potential. Interestingly, decreased plasma levels of miR-107 correlated with abnormal cortical anatomy, common to AD patients, while injecting miR-107 mimic reversed spatial memory impairment, decreased phosphorylated Tau levels, and Aβ neurotoxicity (Shu et al., 2018; John et al., 2020).

MitomiR-34a was recently found to affect mitochondrial metabolism contributing to declining spatial memory (Sarkar et al., 2016). In support to the significant potential of mitomiRs in AD, miR-181a is recently reported to affect mitochondrial glucose metabolism and increase mitochondrial dysfunction, while clinically it is upregulated in MCI patients' plasma and has been reported as a promising early diagnostic marker to predict progression to AD (Ansari et al., 2019).

Table 4 illustrates updates about the recently discovered mitomiRs and their potential role in AD progression.

**Table 4:** Mitochondrial miRNAs in relation to mitochondrial dysfunctions.

**miRNA**	**Role**	**Altered level**	**References**
miR-34a	Allocated in the mitochondria and Promotes apoptosis and dysfunction of mitochondria	Upregulated	Sarkar et al., 2019
miR-107	Decreased mitochondrial functions and morphological changes	Downregulated	Rech et al., 2019
miR-126	Reduces aerobic respiration	Upregulated	Tomasetti et al., 2014
miR 23a/23b	Affects mitochondrial biogenesis	Downregulated	Tang, 2016
miR-132	Reduces aerobic respiration	Downregulated	Weinberg et al., 2015
miR-181a	Localized in mitochondria. Promotes apoptosis and dysfunction of mitochondria	Upregulated	Giuliani et al., 2018
miR-762	Reduces ATP production	Downregulated	Yan et al., 2019
miR-210	Clinical marker for MCI and AD Targets mitochondrial iron sulfur cluster homolog	Upregulated	Siedlecki-Wullich et al., 2019
miR-424	Suppression of ATP levels and mitochondrial integrity	Upregulated	Duarte et al., 2014
miR-425	Mitochondrial dysfunction and increased ROS production	Upregulated	Hu Y.-B. et al., 2019

## AD, Sleep Disorders and Circadian Rhythm

### Sleep: The Complex Process of Circadian Rhythm in Action

Circadian rhythms are 24 h cycles that maintain homeostasis in different body tissues. Circadian rhythm is controlled by the suprachiasmatic nucleus (SCN) which is found in the hypothalamus, and it synchronizes multiple functions as sleep/wake cycle, metabolism, thermoregulation, and hormonal regulation. This molecular clockwork involves genetically encoded autoregulatory feedback loops that provide a 24-h period of circadian oscillation (Park et al., 2020).

The core loop of this molecular clock is driven by a heterodimeric transcriptional activator that is composed of two clock genes: circadian locomotor output cycle kaput *(CLOCK)* and brain–muscle–arnt-like protein 1 *(BMAL1)*. These heterodimers accelerate E-box-mediated transcription and increase gene expression of negative regulators; [Periods *(PERs: PER1, PER2, and PER3)* and Cryptochromes *(CRYs: CRY1 and CRY2)*] and circadian output genes. Expressed and dimerized *PER: CRY* represses the transcriptional activity of CLOCK:BMAL1, hence, downregulate their own gene transcription. It takes 24 h to complete such loop cycle and the accurate generation of 24 h cycles is regulated by post-translational modifications that includes phosphorylation, ubiquitination, and acetylation. Majorly, the phosphorylation of *PER* proteins by casein kinase Iε *(CKI* ε*)* and glycogen synthase kinase-3β *(GSK-3* β*)* promotes *PER* nuclear translocation, thereby provide proper completion of the cycle (Eide et al., 2005).

The SCN also contains gamma-aminobutyric acid (GABA) and arginine vasopressin (AVP) neurons that send inhibitory signals to the paraventricular nucleus of the hypothalamus (Reghunandanan and Reghunandanan, 2006). This activates melatonin secretion by the pineal gland and when it binds to MT1 and MT2 receptors, it inhibits the firing of the SCN. Hence, melatonin promotes sleep and resets the circadian pacemaker (Aulinas, 2000).

### AD and Sleep Disturbances

Circadian rhythms that regulate sleep gradually weaken with aging causing disturbances in sleep quality and cognitive alterations. However, circadian rhythms are markedly disturbed in AD all through the disease course. Day-time agitation, night insomnia, restlessness and sun-downing are among the characteristic changes observed in AD and they worsen with AD progression to affect around 42–52% of AD patients. Such sleep changes are majorly attributed to disruptions in the precise cascade of circadian rhythm (Todd, 2020).

AD is often associated with changes in the physiological parameters of sleep that include decrease in total sleep time and efficiency, prolonged sleep time stage 1 and stage 2 sleep, lesser time in deeper sleep, increased REM sleep latency and decreased REM sleep, together with decreased density of eye movement activity (Weldemichael and Grossberg, 2010).

Another major disruption of AD-sleep disturbance is the “Sundowning.” This condition refers to a delirium-like status usually occurring at late-afternoon and till dawn. Behavioral components of sundowning can include loud vocalization, wandering, physical aggression, maladaptive physical behaviors, and overall agitation. The prevalence of sundowning in AD ranges from 12 to 25%. The increased frequency for AD agitation at night imply that these chronobiologic changes are affected by disruptions in “the timing” of physiological events (Weldemichael and Grossberg, 2010).

### Causes and Deeper Look to miRNAs Role

The main reason for the sleep-wake disrupted cycle in AD is related to alterations in the suprachiasmatic nucleus (SCN) and melatonin secretion. Moreover, expression of MT1 receptors is decreased in the SCN of AD patients resulting in reduced melatonin production and disappearance of melatonin rhythm (Todd, 2020). Till date, the molecular mechanisms underlying these disturbances are still not fully resolved which leave a tight margin for effective therapeutic intervention. Interestingly, collected data from both human and animal studies show that sleep disturbances are not only a consequence of AD progression, but may also precede AD symptoms onset and may contribute to AD pathology through affecting Tau and Aβ deposition and clearance from the brain (Musiek et al., 2015). However, despite the high frequency of sleep disturbances during AD course, there is an obvious lack of data that specifically discusses the molecular basis of AD-sleep disorders, apart from other AD-disruptions.

In a recent study, deletion of the master gene BMAL1 abrogated all circadian functions, leading to complete loss of day-night rhythmicity of sleep (Musiek et al., 2015). Simultaneously, sleep deprivation was found to change the expression of clock genes and BMAL1/CLOCK heterodimers binding, thus altering clock function.

Growing evidence now confirm that mature miRNAs are crucial for the fine-tuning of circadian rhythm regulations that also include sleep (Table 5 enlists some of the miRNAs reported to regulate Circadian Rhythm in different tissues).

**Table 5:** MicroRNAs regulating core clock components.

**miRNA**	**Affected clock component**	**References**
miR-449a	*PER* gene	Hansen et al., 2011
miR-103		Chen et al., 2019
miR-125a-3p		Ma et al., 2020
miR-192		Garufi et al., 2016
miR-194		Nagel et al., 2009
miR-24		Oyama et al., 2017
miR-29a/b/c		
miR-30a		
miR-34a		Ma et al., 2020
miR-211	*Ebox, NPAS2, CLOCK* Genes	
miR-107		
miR-124		
miR-141		Na et al., 2009
miR-17-5p		Gao et al., 2016
miR-182-5p		Ma et al., 2020
miR-19b		Na et al., 2009
miR-199b-5p		Yuan et al., 2020
miR-206		Garufi et al., 2016
miR-10	BMAL1 Gene	Zheng et al., 2021
miR-135b		Zheng et al., 2021
miR-142-3p		Tan et al., 2012; Shende et al., 2013
miR-155		Shende et al., 2011
miR-211		Ma et al., 2020
miR-27b-3p		
miR-494		Shende et al., 2011
miR-106b	*CRY* Genes	Zheng et al., 2018
miR-181a,d		Na et al., 2009
miR-185		Lee et al., 2013
miR-340		Zheng et al., 2018
miR-219	*ROR* output genes	Ma et al., 2020
miR-142-3p		Shende et al., 2013
miR-125a-3p	*(CKI ε) and (GSK-3 β)*	Zheng et al., 2018
miR-181a	*RORα Genes*	Zheng et al., 2018
miR-27a-3p		Zheng et al., 2018
miR183-5p		Dambal et al., 2015
miR-450-5p		Zheng et al., 2018
miR-19b		Linnstaedt et al., 2020
miR-503-5p		Zheng et al., 2018
miR-126a-3p	*E4BP4, DBP Genes*	Cheng et al., 2007

Moreover, although differential expression of multiple miRNA panels has been detected in AD, it is of high interest that consensus miRNAs that regulate key genes in AD pathogenesis, are also involved in sleep-circadian disorders. For example, miR-219 is reported to be overexpressed in postmortem brain tissues of AD patients and interestingly, miR-219 regulates Tau phosphorylation and targets GSK-3 which is vital for phosphorylating PER genes (Kinoshita et al., 2020). Moreover, miR-219 modulates CLOCK-BMAL1 complex. Similarly, miR-132 modulates GSK-3 and Tau Phosphorylation and it is downregulated in AD neurons (El Fatimy et al., 2018).

MiR-125a, miR-125b, miR-146a have high diagnostic potential to predict AD progression, where they modulate Tau hyperphosphorylation (Nagaraj et al., 2019), inflammatory responses and autophagy in microglia and astrocytes, while regulating PER genes in the circadian clock (Lee et al., 2016; Liang et al., 2021). MiR-125 also regulates the cholinergic functions via modulating CLOCK gene. Meanwhile miR-146a is associated with short sleep (Davis et al., 2007; Karabulut et al., 2019) and shows rhythmic expression. It is worth mentioning that miR-146a shows remarkable potential as a diagnostic AD biomarker (Siedlecki-Wullich et al., 2019). Differential expression of miR-34a has also been detected in the brains and blood of AD patients, where it regulates genes involved in memory formation, amyloid precursor protein metabolism and Tau phosphorylation (Sarkar et al., 2016). Intersecting with these functions, miR-34a also regulates PER1 and PER2 genes. MiR-29 family members are established as potential indicators of AD status. In line, miR-29a, b, c are also involved in regulating PER genes, β-secretase (BACE1) mRNA and Aβ accumulation (Müller et al., 2016). Similarly, miR-107 is down regulated in the temporal cortex and plasma of AD patients and it targets both *CLOCK* gene and *BACE1* expression.

MiR-155 is among the most well-studied microRNAs in AD-neuroinflammation. The high expression level of miR-155 in 3xTg AD animal model is accompanied with hyperactivation of microglia and astrocytes, to trigger inflammatory mediators. Moreover, miR-155 contributes to AD through activating different T cell functions during inflammation. Clinically, miR-155 is upregulated in human AD brains and it aggravates neuroinflammation (Kou et al., 2020). Similarly, miR-181 family impaired levels are also repeatedly reported in AD and are linked to accumulated plaque formation in the temporal complex and regulation of inflammation cytokine TNF-α and IL-6 (Kou et al., 2020). Both miR-155 and miR-181 have been linked to sleep disorders in AD as well as other neurodegenerative disorders (Slota and Booth, 2019).

Despite the scarcity of studies exploring miRNAs in AD- associated sleep disorders, the previously mentioned data strongly suggests the association between miRNAs dysregulations and sleep disorders together with other AD molecular disturbances. Clarifying this interconnected network with more studies, might unravel the molecular basis of sleep disturbances in AD and provide novel approaches for better and earlier management of AD.

## Pain: the Underestimated Companion in Alzheimer's Disease?

Alzheimer's is often co-morbid with chronic pain, where chronic pain prevalence is around 45.8% in AD (van Kooten et al., 2016). Pain that results from damage of body tissues is classified as nociceptive pain, while pain resulting from direct consequence of a lesion or disease that affects the somatosensory system” is classified as neuropathic pain (NP) (Merskey et al., 1994). Over the course of AD, AD patients can experience both nociceptive and neuropathic pain. And while, peripheral neuropathic lesions do not always cause chronic pain, central neuropathic pain is often chronic (Husebo et al., 2016).

Due to their inability to communicate and express their needs, pain feeling is often overlooked in AD patients. However, pain is more prevalent with severe dementia (Rajkumar et al., 2017), and its intensity is positively correlated with dementia severity (Whitlock et al., 2017). Impaired pain perception that is mediated by central nervous system is crucial in chronic pain. Simultaneously, central sensitization of pain processing pathways impacts cognition and emotional processing. These pathological interactions imply the presence of deeper link between chronic pain and AD progressiveness.

The “locus coeruleus” (LC) modulates pain and is a key player in chronic pain processing (Taylor and Westlund, 2017). It also innervates most brain areas and is the principal site of norepinephrine (NE) synthesis and neurotransmission in the CNS. The mechanistic processing underlying chronic pain is a complex issue that still needs to be resolved, nevertheless when experienced with AD.

Increasing evidence now shows that chronic pain and AD share disrupted function and structure of the LC. Moreover, both human and animal reports show that chronic pain induces microglial activation and neuroinflammation in hippocampus, anterior cingulate cortex, amygdala, nucleus accumbens, thalamus, and sensory cortex (Cao et al., 2019). This is associated with elevated release of inflammatory cytokines; TNF-α, IL-6, IL-1β that trigger disruptions in synaptic remodeling, brain connectivity and network function. Interestingly, microglial activation and neuroinflammation are found to precede cognitive decline in AD patients, implying its participation in aggravating AD disease (Fan et al., 2017). The chronic activation of microglia induces synaptic loss, and this occurs in both AD and chronic pain. Moreover, the prolonged exposure to amyloid depositions, has also been found to activate microglia, resulting in excessive secretion of synaptic-toxic cytokines, and tau accumulation that eventually cause synaptic loss and neuronal death (Cao et al., 2019). Besides microglial activation, disrupted autophagy process has been increasingly reported as a significant contributor to both chronic pain and AD (Yin et al., 2018).

**Figure 4:**
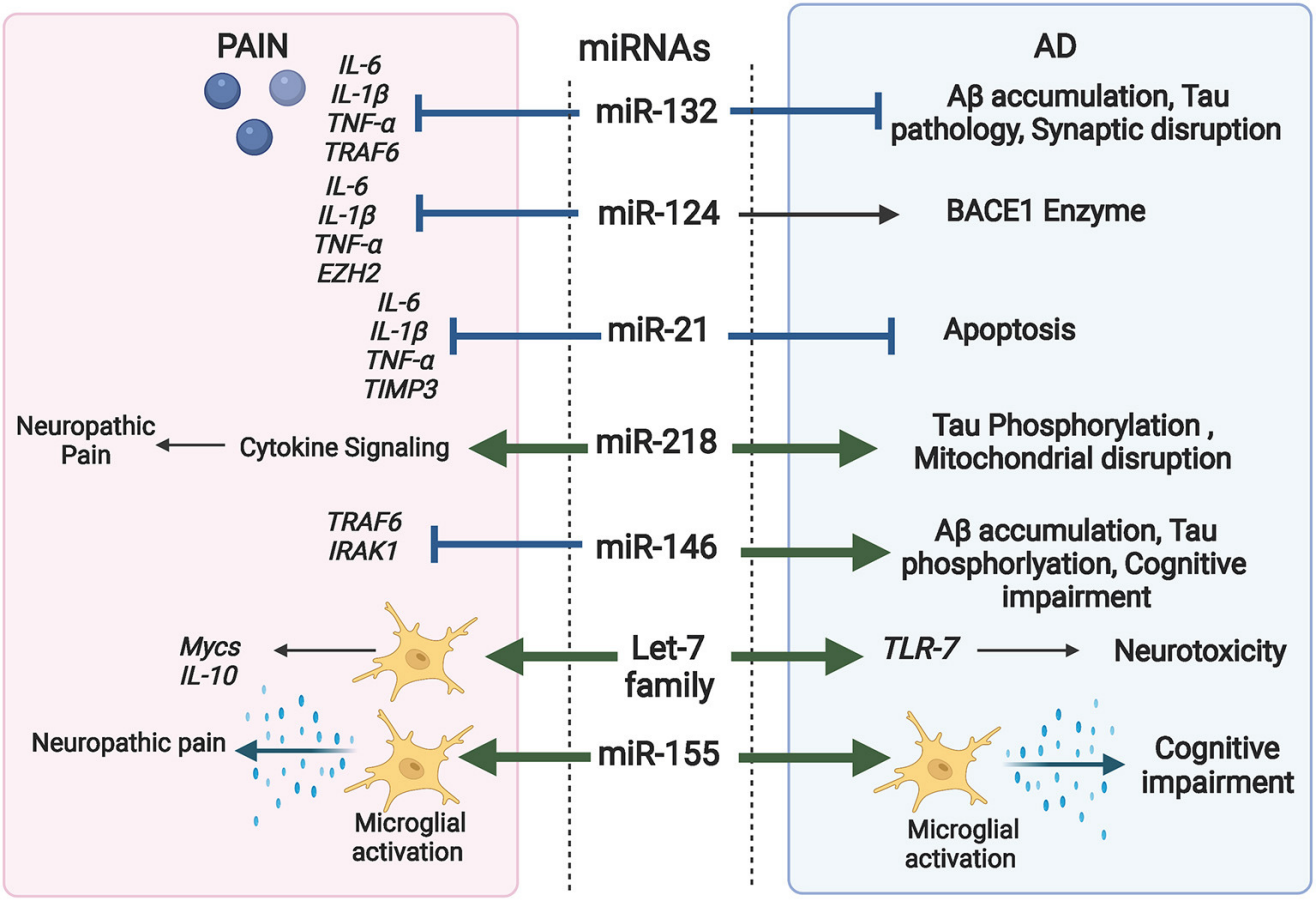
Comprehensive illustration of the overlapping role of some miRNAs in both AD and Pain and their targeted genes. (Created with BioRender.com).

It is established that miRNAs are key players in modulating macromolecular complexes in neurons, glia, and immune cells. They regulate signals interconnecting neuro-immune network in the pain pathway and are crucial modulators of inflammation and autophagy pathways; two major factors in AD progression as well as chronic pain (López-González et al., 2017; Bernaus et al., 2020). As a result, miRNAs are now considered as significant “master switches” in chronic pain and AD. Figure 4 highlights the overlapping role of some miRNAs in AD and pain.

For instance, miR-132 participates in regulating inflammation and is a negative regulator of the inflammatory response in PC12 cells. IL-1β, IL-6, TNF-α and TNF receptor associated factor 6 *(TRAF6)* are potential targets of miR-132 (Kou et al., 2020). Recently upregulation of miR-132 in WBCs of patients were associated with chronic neuropathic pain (Leinders et al., 2016). Interestingly, miR-132 is significantly reduced in the brains of AD patients and deletion of miR-132 in mice hastened Aβ accumulation, and tau pathology via modulating the synaptic proteins (Xu N. et al., 2019). In line, the down regulation of miR-132 was found to inhibit the level of hippocampal acetylcholinesterase *(AChE)*, impacting both cognitive function and synaptic plasticity. The downstream molecules responsible for miR-132 actions involve both p250 GTPase Activating Protein *(p250GAP)* and Methyl CpG-Binding Protein 2 *(MeCP2)* (Ye et al., 2016).

MiR-155 is one of the most prominent miRNAs that are differentially expressed in AD serum and brain. Recent reports confirm that miR-155 has significant impact on development of pain and pain hypersensitivity, where up-regulation of miR-155 is accompanied by enhanced activation of microglia and consequent production of inflammatory mediators (Kou et al., 2020). Moreover, miR-155 is a significant regulator of neuropathic pain via targeting serum and glucocorticoid regulated protein kinase 3 *(SGK3)*, an important protein involved in phosphorylation cascades (Liu et al., 2015).

Similarly, Let-7 is critical for maintaining microglial function in inflammation-mediated injury (Roush and Slack, 2008). Let-7a inhibits the expression of inflammatory cytokines via activation of apoptosis signal-regulating kinase 1 (ASK1), IL-10 and *Mycs* in microglia (Song and Lee, 2015). Meanwhile, let-7 miRNAs are found to be differentially and specifically released in CSF of AD patients (Derkow et al., 2018). MiR-124 is another brain-enriched miRNA involved in the regulation of neural development. Increasing reports spot its role as a remarkable alleviator of neuropathic pain, via inhibiting (IL-6, IL-1β, and TNF-α) protein expressions and direct targeting of Enhancer of zeste homolog 2 *EZH2* gene (Zhang et al., 2019). Moreover, miR-124 regulates BACE1 enzyme and is found to decrease gradually with AD progression (An et al., 2017). Most importantly, updated studies are now spotting miR-124 as a therapeutic target for its role in modulating inflammation in the central nervous system and brain injuries (Xu et al., 2021).

MiR-21 is another interesting miRNA regulator of pain that is increasingly reported as a significant contributor against AD progressive events. MiR-21 can inhibit cell apoptosis induced by Aβ_1−42_ via modulating cell death protein 4 *(PDCD4)*/phosphatidylinositol 3-kinase PI3K/AKT/GSK-3β pathway in the CNS (Feng et al., 2018). Meanwhile circulating miR-21-5p was significantly upregulated in the plasma of AD patients and was negatively correlated to cognitive impairment (Giuliani et al., 2021). Simultaneously, disturbed level of miR-21 was observed in diverse neuropathic pain models (Zhong et al., 2019) where it targets metalloproteinase-3 *(TIMP3)* and chemokines C-C motif ligand 1 *(CCL1)* that consequently evoke cytokine production of TNF-α, IL-1β, and IL-6 and aggravate neuroinflammation.

MiR-146a-5p is reported to modulate immune response and reduce inflammation by targeting both *TRAF6* and interleukin-1 receptor-associated kinase 1 *(IRAK1) IRAK1* in macrophages, and astrocytes. Lu et al. (2015) reported the protective effect of miR-146a against SNL-induced neuropathic pain by suppressing *TRAF6* signaling in the spinal cord (Lu et al., 2015). Meanwhile, multiple studies linked miR-146a-5p with cognitive deterioration, where its upregulation in the CNS is associated with increased expression of Aβ, Taup38, and Reactive oxygen species (ROS) through targeting *MAPK* signaling (Alexandrov et al., 2014).

One of the emerging and promising miRNAs in both AD pathology and chronic pain is miR-218. Recently miR-218 upregulation was found to contribute to AD progression by enhancing Tau phosphorylation and disrupting mitochondrial respiratory chain through modulating *Wnt* signaling pathway (Gugliandolo et al., 2020; Wu et al., 2020). Meanwhile, downregulation of miR-218 was proved effective in suppressing central neuropathic pain via regulating cytokine signaling (Li and Zhao, 2016).

Elucidating the regulatory role of miRNAs on pain sensitization, neuropathic pain and the interplay with cognition and behavior alterations in AD, can unleash new resolutions on the pathophysiology of chronic pain in AD. Considering the common role of miRNAs in regulating chronic pain and its possible contribution in worsening cognitive impairment, miRNAs might also provide a prognostic tool to predict susceptible AD patients.

### MicroRNAs as Diagnostic Biomarkers of AD

Current AD diagnostic markers and methods are applicable in the late stages of AD. Ultimately, they can be classified into (1) Neuropsychological tests: which are cognitive assessments used to quantitatively assess the degree of cognitive impairment and its progression over time. This method, however, has limited specificity and sensitivity and is majorly affected by patients' educational levels. (2) Neuroimaging examination: such as Magnetic resonance imaging (MRI) and fluorodeoxyglucose (FDG)-positron emission tomography (PET) that monitor the pathological and functional alterations before severe appearance of cognitive impairment (Calvillo and Irimia, 2020). However, this method is hugely limited by the high cost.

Recent trials reported the high diagnostic potential of Amyloid and Phosphorylated Tau /Aβ ratio in CSF samples. Yet again, this technique is highly invasive and requires well-trained personnel for sample acquisitions. The detection of neuro filament light chains (NFL) in biological samples is emerging as good neuronal biomarkers, however, techniques and kits for reliable detection are still limited, and more research studies are needed to explore their ability to differentiate between different neurodegenerative diseases (Gaetani et al., 2019).

In the previous decade, the reported differential expressions of miRNAs in animal models of AD have opened the field to unleash the potential of miRNAs as promising diagnostic biomarkers for multiple neurodegenerative diseases. Moreover, continuous reports show that specific miRNAs are detected in the biofluids of AD patients with different levels from normal controls, along with their correlation to AD observed pathological and cognitive changes (Wei et al., 2020). A point that can be optimized to monitor AD progression. Furthermore, circulating miRNAs that are collected from serum or plasma resist environmental degradation and can provide a cheaper and less-invasive diagnostic means, compared to neuroimaging and CSF examinations. Ongoing research continuously reveals the remarkable diagnostic potential of miRNAs in AD. Table 6 presents an updated summary for different miRNAs that have shown promising potential as AD diagnostic markers.

**Table 6:** MicroRNAs with diagnostic potential in human AD studies.

**miRNA**	**Sample**	**Expression in AD**	**Studied cohort**	**Specificity/sensitivity**	**References**
miRNA-483-5p	Plasma	Increased	20 MCI 20 AD 20 Healthy controls	95, 90%85, 90%	Sabry et al., 2020
miR-26a	Serum	Decreased	121 AD, 48 HC	57, 85%	Guo et al., 2017
miR-34a	Plasma CSF	Increased Decreased	25 AD, 27 HC 10 AD, 10 HC	74, 84%	Cosín-Tomás et al., 2017
miR-125b	Serum	Increased	105 AD, 155 HC	68, 80%	Tan et al., 2014a
miR-181a	Serum	Decreased	121 AD, 86 HC	73, 72%	Ansari et al., 2019
miR-181c	Serum	Decreased	150 HC, 105 AD	64, 75%	Manzano-Crespo et al., 2019
miR-206	Serum	Decreased	66 MCI, 33 HC	100, 70%	Xie et al., 2015
miR-342-3p	Serum	Decreased	158 AD, 155 HC	70, 81	Tan et al., 2014b
miR-34c	Blood mononuclear cells	Increased	110 AD, 123 HC	74, 84%	Bhatnagar et al., 2014
miR-133b	Serum	Decreased	98 AD, 105 HC	74.3, 90%	Yang et al., 2019
miR-433a	Serum	Decreased	32 AD, 12 HC	0.82 AUC	Wang and Zhang, 2020
miR-103	Plasma	Decreased	120 AD, 120 PD, 120 HC	84, 80%	Wang J. et al., 2020
miR-106	Serum	Decreased	56 AD, 50 HC	62, 94%	Madadi et al., 2020
miR-9	Serum	Decreased	36 AD female patients 38 HC females	Not available	Souza et al., 2020
miR-29cmiR-19b	Serum	Decreased	45 AD, 40HC	Not available	Wu et al., 2017
miR-455-3p	Serum	Increased	11 AD, 18 MCI, 20 Healthy	AUC 79%	Kumar et al., 2017
miR-let-7b	CSF	Increased	41 memory complaints, 36 MCI, 17 AD patients	Addition to total and *p- tau* panel led to improved AUC to 91.6%	Liu et al., 2018

Spotting the most prominent miRNAs, recent studies have investigated the predictive potential of miRNAs in longitudinal studies over time. In an updated study, plasma miR-206 level stands out as a good prognostic biomarker to monitor MCI progression to AD over a period of 5 years (Kenny et al., 2019). In the same context, Ansari et al. (2019) showed the ability of miR-181a and miR-146a blood levels to predict whether MCI would progress to AD or remain stable after 2 years monitoring (Ansari et al., 2019).

Specific single miRNAs have shown consistent differentially expressed levels in various AD samples. For instance, Persistent miR-26b upregulation is reported in both serum and whole blood AD samples (Galimberti et al., 2014). Yang et al. (2018), recently spotted the diagnostic ability of exosomal miR-384, as its expression in serum of AD and non-AD patients differ significantly. Furthermore, serum level of exosomal miR-384 showed potent differential diagnostic ability for AD and Parkinson's dementia, as well as for AD and vascular dementia, with sensitivity/specificity indices of 97.2/100% and 99.1/100%, respectively (Yang et al., 2018).

One of the most emerging and promising miRNAs in AD diagnosis is miR-455-3p. Where serum miR-455-3p is upregulated in AD patients as compared to both MCI subjects and healthy controls. Interestingly, this finding is also confirmed in fibroblast cells, postmortem AD brains examination at different Braak stages (Kumar et al., 2017; Kumar and Reddy, 2018), as well as in CSF samples of sporadic AD subjects (Kumar and Reddy, 2021). Analyzing the mechanistic effects of miR-455-3p in AD showed that *APP* is a validated target of miR-455-3p. Whereas, elevated levels of miR-455-3p help to alleviate Aβ toxicity, improve mitochondrial dynamics and synaptic activity (Kumar and Reddy, 2019).

Another important concept is the utilization of miRNA panels of multiple miRNAs as a signature for AD. This can provide higher accuracy, specificity, and sensitivity. In this context, miR-181c, miR-92a-3p, and miR-210-3p showed remarkable ability to differentiate between AD and healthy controls. Moreover, this signature panel showed promising results in predicting MCI progression to AD after monitoring patients for 1 to 11 years (Siedlecki-Wullich et al., 2019). Additionally, a serum 9-miNRA signature panel including; miR-26a-5p, hsa-miR-181c-3p, hsa-miR-126-5p, hsa-miR-22-3p, hsa-miR-148b-5p, hsa-miR-106b-3p, hsa-miR-6119-5p, hsa-miR-1246, and hsa-miR-660-5p was recently reported to differentiate between AD and healthy controls with an AUC ROC reaching 85%, in a study that comprised one of the biggest cohorts of AD patients (Guo et al., 2017).

Although, miRNAs possess the properties of good diagnostic tools, miRNA use is still faced by several limitations. Namely, the parameters used for groups classification, subjects' inclusion and exclusion criteria, miRNAs extraction and quantification methods and reliable reference genes, vary widely among different labs. This is why finding a method to standardize miRNAs isolation and quantification protocols, as well as recruiting wider and more diverse populations can provide deeper knowledge about miRNA diagnostic potential.

### The Therapeutic Potential of miRNAs in AD

The cumulative knowledge on miRNAs functions, strongly implies their potential as an emerging therapeutic possibility for multiple AD pathologies. Generally, therapeutic intervention using miRNAs can take three approaches: (1) Using natural or synthetic compounds to modulate the expression of miRNAs (2) inhibiting the function of a particularly targeted miRNA using complementary single stranded antisense oligonucleotide (ASO). (3) Readjusting the expression of the targeted miRNA using miRNA mimics. So far, several trials have been conducted to adjust miRNA expression in AD animal models as well as cell lines.

Numerous studies used natural or synthetic compounds to readjust miRNAs expressions. Among the most characteristic trials, Osthole is reported to modulate the expression of miR-9 and miR 101a-3p in APP/PS1 mice, SHSY-5Y cells as well as neural stem cells. Consequently, this resulted in decreased cellular apoptosis, improved cell growth, improved learning and memory capacities, together with prevention of Aβ aggregation (Li et al., 2017). Berberine was also reported to remodulate the expression of miR-188 in BV2 cells, resulting in remarkable inhibition of Apoptosis (Chen et al., 2020). The Chinese herbal Tiaoxin recipe was recently reported to inhibit miR-34a expression in APPswe/PS1ΔE9 mouse AD model leading to decreased Aβ aggregation (Hu Y.-R. et al., 2019). A combination of resveratrol and curcumin was also successfully reported to readjust the expression of cortical let-7c in rats and PC-12 cells, which led to significant reduction of neuroinflammation along with readjustment of β-secretase and APP expressions (Zaky et al., 2017). In the same context, Sun et al. (2020), recently reported the ability of Dexmedetomidine to upregulate miR-129 in NIH Swiss mice, which led to cognitive improvement through targeting *YAP1* gene (Sun et al., 2020).

The continuous advances in drug delivery and biotechnology help in the rapid progress toward specific miRNA targeting and delivery. Interestingly, a recent study that used engineered exosomes to deliver miR-29 in a rat model of induced Aβ pathology, showed significant improvement of memory deficits (Jahangard et al., 2020). Meanwhile, photoactivation of targeted miRNAs, miRNAs sponges that sequester a specific miRNA and inclusion of miRNAs in labeled liposomes or cubosomes to cross the blood brain barrier are all emerging innovative means that can be optimized in the near future for miRNA delivery (López-González et al., 2017).

## Discussion and Outline

### Current Hopes, Challenges, and Future Perspectives

There is no doubt miRNAs provide a novel opportunity to tackle AD disease, either through their potential as earlier diagnostic markers or through modulating their expression to reinstate the relevant AD target genes.

One interesting point is that along our research, numerous miRNAs apparently act as multi-faceted modulators of AD-interconnected signaling pathways. A point that can be advantageous in re-adjusting the different impaired pathways affected by miRNAs dysregulations. Furthermore, despite their nature as epigenetic modulators that can influenced by environmental and ethnic variations, multiple miRNA panels were repeatedly reported in different AD studies of variable regions and populations. For instance, miRNAs including but not restricted to; miR-155, miR-146a, miR-34a, miR-29, miR-132, miR-483-5p, and miR-181 are reported as differentially expressed in multiple AD samples of Chinese, American, European populations (Cheng et al., 2015; Wei et al., 2020; Huaying et al., 2021; Siedlecki-Wullich et al., 2021), as well as emerging studies from Africa and middle eastern regions (Sabry et al., 2020). At the same time, these miRNAs have also been introduced as crucial modulators of Aβ and Tau pathologies, inflammatory, mitochondrial, and synaptic dysfunctions that accompany AD progression.

An emerging hot topic in AD pathology is the role of mitochondrial miRNAs in mitochondrial dysfunction and mitophagy that are highly likely to precede actual AD neuronal damage (John et al., 2020). And while recent studies show miR-7, miR-155, miR-210 and miR-125 as crucial in mitochondrial impairments accompanying cancer (Ortega et al., 2020), we are still scratching the surface concerning mitomiRs in “inflamm-aging “axis and AD.

Another hot topic in AD dilemma is the promising role of lactate level in astrocytes and its possible effect on reinstating cognitive decline of AD. Updated studies spot the role of miRNAs; miR-34a (Sarkar et al., 2016), exosomal miR-137 (Thomas et al., 2020) and miR-124-3p (Xu S.-Y. et al., 2019) in controlling astrocytes lactate shuttle in different brain disorders and the consequent impact of adjusting their levels on cognitive improvement. Further future studies can definitely elucidate more facts about miRNAs role in astrocyte metabolism during AD and unravel ways to use this knowledge.

That being said, it is inevitable to state that till date, miRNAs bench studies are still faced by several obstacles that need to be resolved before being capable of efficiently serving AD clinical applications.

For a beginning, till now, there are limitations in comparing miRNA studies due to the basal differences in AD stages of the recruited patients and unclear specificity to AD. Moreover, communication difficulties with AD patients and their caregivers, comprise a major challenge by itself to recruit bigger populations of AD patients. To improve comparability in miRNAs studies, classifying the recruited patients in comparable groups according to their disease stages, followed by correlating their pathology with their miRNA's profiles can be more reliable.

Another challenge is the scarcity of data originating from middle and low-income countries and the relatively small populations size in AD clinical studies that majorly originate from well-developed countries. This can be resolved by enhancing the means of reaching out to affected communities, performing more longitudinal studies, together with utilizing advanced screening techniques, and raising awareness about the critical significance of participating in AD studies.

On the technical level, an important challenge in miRNAs studies, is the wide variability in sample collection, storage, standardization against reference controls, and analytical protocols (Mushtaq et al., 2016). Multi-center comparisons and universal standardization of the used techniques would to a large extent improve miRNAs reliability.

Indeed, efficient use of miRNAs for AD diagnosis, still needs to be preceded by precise standardization of sampling and quantification protocols and proper staging of AD. It might also be useful to rely on multiple panels of miRNA or combinations of miRNAs together with other fluid biomarkers. Progresses in this aspect are continuously accomplished. Interestingly, some registered clinical trials are already running for example: (NCT03388242) that is using a combination of microRNAs and proteins with different expression patterns to distinguish between normal control, MCI and AD patients (Zuo, 2019).

Therapeutically, miRNAs use is challenged by some issues. Firstly, miRNAs target multiple genes and hence, manipulating one miRNA can result in unwanted effects on other sides (Siedlecki-Wullich et al., 2021). However, advances in *in silico* analysis and neuroinformatics can help face this problem. Besides, conducting more studies from diverse populations can contribute to getting more precise data that guide researchers to the proper beginning concerning their targeted population. Moreover, identification of the individual roles of specific miRNAs, as well as the collective role of multiple miRNAs in AD should be explored.

Another challenge is the difficulty of efficient miRNA delivery to the brain. However, the increasing advances in nanotechnology (Abdel-Mageed et al., 2021) and targeted delivery of biological materials have been successfully reported in some AD animal models.

Interestingly, miR-155 is already entering a clinical phase trial as a therapeutic compound registered by ©Miragen Therapeutics against Amyotrophic lateral sclerosis (Chakraborty et al., 2021). We believe, the near future can witness similar clinical successes to face the challenging pathologies of AD.

## Author Contributions

NA and FN drafted the manuscript. AZ and AB performed critical editing. NA, MA, and FN participated in constructive outline, discussions, and editing. All authors read and approved the final manuscript.

## Funding

This work was partially funded by the Science and Technology Development Fund (STDF), Egypt (Grant Number 39436) Youth Research Project.

## Conflict of Interest

The authors declare that the research was conducted in the absence of any commercial or financial relationships that could be construed as a potential conflict of interest.

## Publisher's Note

All claims expressed in this article are solely those of the authors and do not necessarily represent those of their affiliated organizations, or those of the publisher, the editors and the reviewers. Any product that may be evaluated in this article, or claim that may be made by its manufacturer, is not guaranteed or endorsed by the publisher.

## Abbreviations

BACE1: Beta-Secretase 1
HEY2: Hairy/Enhancer-Of-Split Related With YRPW Motif Protein 2
APP: Amyloid beta precursor protein
MAPK: Mitogen-activated protein kinase
DUSP6: Dual specificity phosphatase 6
PPP1CA: Protein Phosphatase 1 Catalytic Subunit Alpha
PTBP2: Polypyrimidine Tract Binding Protein 2
*MeCP2*: Methyl-CpG Binding Protein 2
PTEN: Phosphatase And Tensin Homolog
*Fyn*: Proto-oncogene tyrosine-protein kinase Fyn
ERK1: extracellular signal-regulated kinase 1
ERK2: extracellular signal-regulated kinase 2
CREB: cAMP response element-binding protein
BDNF: Brain-derived neurotrophic factor
*EphB2*: Ephrin type-B receptor 2
*GRIA2*: Glutamate ionotropic receptor AMPA type subunit 2
*GRIN2B*: Glutamate Ionotropic Receptor NMDA Type Subunit 2B
*Syt-1*: Synaptotagmin1
*Syt-1A*: Syntaxin 1A
*NPTX1*: Neuronal Pentraxin 1
*AMPAR*: α-amino-3-hydroxy-5-methyl-4-isoxazolepropionicacid receptor
*GRIN2A*: Glutamate Ionotropic Receptor NMDA Type Subunit 2A
*EPHA4*: Ephrin type-A receptor 4
*Nrn1*: Neuritin 1
*Arpc3*: Actin Related Protein 2/3 Complex Subunit 3
*CPLX1*: Complexin 1
*CPLX2*: Complexin2
*STIM2*: Stromal Interaction Molecule 2
REST: RE1 Silencing Transcription Factor
*CX3CL1*: C-X3-C Motif Chemokine Ligand 1
CX3CR1: C-X3-C Motif Chemokine Receptor 1
*NPAS2*: Neuronal PAS Domain Protein 2
*DBP*: D-Box Binding PAR BZIP Transcription Factor
*Mycs*: MYC Proto-Oncogene
*YAP1*: Yes1 Associated Transcriptional Regulator
*C-MAf*: C musculoaponeurotic fibrosarcoma
*CCM1*: Cerebral cavernous malformations 1
*NOX2*: NADPH oxidase 2
*NOX4*: NADPH oxidase 4
*C/EBP-*α: CCAAT/enhancer-binding protein alpha
TREM2: Triggering receptor expressed on myeloid cells 2.
